# The roles of attachment and resilience in perceived stress in medical students

**Published:** 2018-11-12

**Authors:** Galilee Thompson, Andrew Wrath, Krista Trinder, G. Camelia Adams

**Affiliations:** 1 University of Saskatchewan, Saskatchewan, Canada

## Abstract

**Background:**

Medical students are susceptible to high levels of psychological stress, while being equipped with lower levels of resilience, especially females. Adult attachment is a known risk factor for a broad range of mental health difficulties and poor coping. The purpose of this study is to examine relationship attachment style, perceived stress, and resilience in medical students.

**Methods:**

Data was collected via an online survey using self-report measures from University of Saskatchewan undergraduate medical students (*n* = 188). Attachment was assessed with the Relationship Questionnaire and Experiences in Close Relationships Scale. Resilience and stress were assessed with the Connor-Davidson Resilience Scale and Perceived Stress Scale, respectively.

**Results:**

Approximately half of our sample endorsed secure attachment style (49.4%). Females reported significantly more attachment insecurity, higher attachment anxiety, higher perceived stress, and lower resilience compared to males, as expected. As predicted, attachment anxiety and avoidance were predictors of perceived stress. Mediation analyses supported the hypothesis that resilience acted as a partial mediator between attachment insecurity and perceived stress.

**Conclusion:**

These findings suggest attachment plays a role in perceived stress in medical students. In addition, the role of resiliency in protecting against this effect highlights potential areas for intervention to improve medical student well-being and provides a foundation for longitudinal follow-up.

## Introduction

Medical training is generally regarded as a stress-inducing program of study, where students are known to experience higher levels of stress compared to their peers.^1–3^ Medical students face multiple demands including high expectations, potential liability, on-call responsibilities, and competitive training.^4^ In addition, daily encounters with suffering and death of patients, accumulation of debt, long hours studying large volumes of content, and working in cadaver labs have been identified as particularly stressful.^5,6^ As a result, compared to age-matched students, medical students report a decline in life satisfaction by graduation.^7^ Similar findings show that more than 50% of medical students report burnout based on self-report measure of emotional exhaustion, depersonalisation, and low sense of personal accomplishment.^8,9^ A recent international meta-analysis showed that more than 25% of medical students experience symptoms of depression and 11.1% have suicidal ideation.^3^ These concerning findings suggest that medical education is a highly demanding teaching environment where students’ vulnerabilities may be tested. Therefore, to guide the development of effective, evidence-based preventive measures and interventions, we need a better understanding of the individual psychological factors that can either predispose or protect medical students in the face of this high degree of stress. Some have suggested that attachment and resilience are those factors.

### Attachment style

Individual attachment is a well-researched descriptor of interpersonal patterns that stems from early childhood interactions with the primary caregiver. These patterns are likely to perpetuate over time and impact close relationships, the ability to cope with stress, and the overall individual’s function and mental health.^10,11^ In fact, numerous cross-sectional studies and several longitudinal studies have shown that attachment insecurity is not only prevalent among individuals with various psychiatric conditions, but can also be a risk factor.^10–12^ Similarly, college students with high attachment anxiety were shown to have poorer mental health.^13,14^ Research has shown a core attachment pattern to be maintained from childhood to adulthood with moderate to high degree of stability (i.e., a trait component).^15^ In addition, attachment-relevant life experiences were also shown to induce changes, suggesting a state-dependent aspect of attachment as well, possibly amenable to interventions.^10^ Adult attachment has been broadly assessed with two types of measures: categorical or dimensional. Categorical measures classify attachment into four styles, being easy to use clinically. Dimensional measures only look at two attachment dimensions (i.e. attachment anxiety and attachment avoidance) being largely used for research purposes due to their improved validity and reliability.^16^

Attachment styles can broadly be categorized as secure or insecure; with insecure being further divided into preoccupied (i.e., anxious), dismissing (i.e., avoidant) or fearful (i.e., disorganized).^16^ The latter is believed to combine both anxious and avoidant features.^16^ Individuals with secure attachment are characterized by a positive sense of self and others, being comfortable with both intimacy and independence, and resourceful in recruiting necessary help in times of need.^10^ In fact, this ability to understand oneself and others accurately, has been considered a major contributor to the individual’s resilience in the face of stress and challenge.^11^ It has been suggested that attachment and resilience are complementary concepts which share similar developmental circumstances, beginning with a healthy childhood and leading to the emergence of adaptive self-esteem and social empathy, through positive connections with others and one’s culture.^17^ Under stressful conditions, resilient individuals and securely attached individuals alike, are still able to experience healthy self-esteem and a sense of safety as well as maintain their social competence.^11^ In fact, the restoration of attachment security has been shown to increase resilience and improve mental health.^11^ In contrast, individuals with anxious attachment have a lower sense of self and a higher sense of others, resulting in a preoccupation with maintaining closeness in relationships as well as a tendency to overexpress needs, that can be perceived as clingy or demanding.^11^ On the contrary, individuals with avoidant style have a higher sense of self and lower sense of others, being generally excessively independent and minimally trusting of others, therefore achieving lower levels of intimacy.^11^ During stressful times, they tend to be self-reliant and to minimize their distress, therefore, having difficulty expressing it and asking for help.^11^ Similarly, dimensional measures of attachment target the levels of attachment anxiety (equivalent to a negative view of self) and attachment avoidance (equivalent to a negative a view of others). Bartholomew & Horowitz have shown these dimensions to have orthogonal characteristics (being perpendicular on each other in a graphic representation), therefore creating four quadrants analogous to the four attachment styles above.^18^ Therefore, both dimensional and categorical measures have received support being emphasized differently according to the clinical or research context.^18^

In summary, researchers have shown parallels between adult attachment and resilience and their importance in the way stress is perceived and the ability to cope with stress.^11^ However, gender might also play a role, being often associated with insecure attachment. In fact, a large international meta-analysis of gender differences in attachment found that females tend to report higher attachment anxiety, while males tend to report higher attachment avoidance compared to their counterparts.^19^

Numerous, but independent studies have emphasized the relevance of either attachment or resilience in understanding the student population. It seems that the frequency of insecure attachment styles among college students is increasing. A recent meta-analysis concluded that college students reported more insecure attachment styles compared to several decades before, despite demographic variables remaining relatively constant.^20^ Similarly, the connection between attachment and resilience has been captured by several studies which found that students with higher anxious attachment had lower resilience.^13,21^ In the current study we want to further expand this line of research by investigating the relationship between attachment, perceived stress, and resiliency in medical students.

### Resilience

Resilience can be defined as the ability to “bounce back” or thrive despite being faced with difficult situations.^22^ Resilience is a trait that appears to be protective against stress and predictive of well-being.^13,22^ For instance, an Australian study of family medical doctors found that resilience was related to personality traits that support success in the demanding, stressful medical environment; thus, relating generally to well-being.^23^ A study of Brazilian medical students found that those with very low resilience had lower quality of life scores and worse perception of their educational environment than those with very high resilience.^24^ Still, resilience appears to be lower in medical students compared to age-matched peers, particularly in females.^2^

### Research purposes

In summary, medical education is a demanding environment where students’ coping with stress and vulnerability towards psychological disturbances need understanding and support. Individual attachment and resilience have been shown to influence this vulnerability in several studies on general population as well as in students. However, we have not found any studies that have investigated the relationship between individual attachment, resilience and psychological stress in medical students.

The present study intends to address these concerns by studying individual attachment and resilience, as well as their relationship to the perceived level of stress in a sample of medical students from the University of Saskatchewan. The study has four aims:

1. To explore the distribution of insecure attachment styles in a sample of medical students.
2. To explore the gender differences with respect to individual attachment, perceived stress, and resilience.
3. To explore the relationship between attachment and the level of perceived stress. In keeping with previous research, we hypothesized that insecure attachment, particularly the anxious attachment dimension, will predict the level of perceived stress.
4. To explore whether resilience mediates the relationship between attachment dimensions and the level of perceived stress. We hypothesized that resilience will mediate the relationships between attachment and the level of perceived stress.

## Methods

### Participants

The project complied with the standards of the University of Saskatchewan Ethics Review Board and received ethics approval. Participants were students enrolled in the College of Medicine, University of Saskatchewan, at the time of the study (2015). we invited all students from classes expected to graduate between the years 2016-2019 to participate in an online survey. Participation was voluntary, and prior to beginning the study, willing participants gave informed consent. We gave all students who volunteered a unique and confidential survey link via email to access and complete all measures in a single online survey between August 1^st^ and September 30^th^, 2015. Participants received $5 gift cards for their participation.

Out of 399 students invited to participate, 198 students enrolled in the study and 188 completed the survey. The final sample was comprised of 77 males (41.2%) and 110 females (58.8%). One participant did not report their gender, and no one selected “other.” The majority of participants were between the ages of 20 and 29 (n = 175, 93.1%) and single (n = 145, 77.1%). Each of the four graduating classes had a similar number of respondents (n = 45-48).

### Measures

For a comprehensive evaluation of attachment we used both a dimensional and a categorical measure. The Experiences in Close Relationships Scale (ECR) is a 36 item self-report measure of adult attachment. It is a well validated measure of the attachment dimensions, attachment anxiety and avoidance.^18,25^ The Cronbach alpha of the avoidance and attachment scales in this study were 0.93 and 0.90, respectively. The Relationship Questionnaire (RQ) is a categorical measure of attachment, which provides the respondent with the opportunity to self-categorize into one of the four attachment styles based on a brief description of each.^26^ Although categorical measures have been considered less reliable for research purposes, they are usually user-friendly and easier to administer in clinical situations. As a result, they have been widely used in studies. In this study, in addition to a dimensional measure of attachment (i.e., ECR), we also chose to include a categorical one (i.e., RQ) to compare the prevalence of attachment style categories from our sample to previously published work. Given that we used only the categorical portion of the RQ, we could not calculate alpha value.

**The Perceived Stress Scale (PSS)** is a 10-item self-report measure of stress with good validity and reliability.^27,28^ The Cronbach alpha in the present study was 0.89.

**Resilience** was measured with The Connor-Davidson Resilience scale (CD-RISC), a 10 item self-report measure with good internal consistency and construct validity that has been previously used to assess resilience in medical students.^2,29^ The Cronbach alpha for the current study was .89.

### Analyses

To address the first objective, we calculated frequencies for each of the four attachment styles. Data of continuous variables were checked for normality and all variables were found to be skewed. With respect to objective two, we used Mann-Whitney U Test to compare genders on each measure. Pertinent to objective three, we used regression analyses controlling for gender and age to determine if attachment dimensions were predictors of perceived stress. The methods of Baron and Kenny (1986) for mediation analysis were followed to address objective four.^30^ we used univariate analyses between all three variables intended to be included in the model. A mediation occurs when the significance of the relationships between the independent variable and dependent variable decreases with the addition of the mediator to a less significant or nonsignificant level (partial and full mediation, respectively).^30^ All analyses were completed using SPSS Version 24.

## Results

Pertinent to our first objective, 162 participants reported their attachment style on the RQ. The majority of participants reported a secure attachment style (*n* = 80, 49.4%), followed by dismissing (*n* = 43, 26.5%), fearful (*n* = 22, 13.6%), and preoccupied (*n* = 17, 10.5%). The distribution of attachment categories in our sample was similar to the order reported by Mickelson et al. in a large US sample from the general population.^31^ They found the most endorsed attachment category to be secure (59%) followed by avoidant (25%) and anxious (11%). While our sample followed a similar pattern, the rates of secure attachment in our sample were lower and the rates of insecure attachment were higher, compared to Mickelson’s data.

Descriptive statistics of questionnaire data are displayed in Table 1, as are the results of gender comparisons (objective two). Females had significantly higher attachment anxiety, higher perceived stress, and lower resilience (all *p* < .05).

**Table 1:** Descriptive statistics and gender differences on continuous variables

Variable	Total Sample Mean (SD)	Males Mean (SD) Median	Females Mean (SD) Median	*p-*value
Attachment avoidance	2.89 (1.00)	2.73 (.852), 2.61	3.00 (1.08), 2.75	.161
Attachment anxiety	3.40 (1.03)	3.10 (1.06), 2.83	3.61 (0.96), 3.52	<.001
PSS total score	14.24 (6.13)	12.92 (5.79), 11.00	15.19 (6.24), 14.00	.013
Resilience	19.75 (5.73)	21.60 (5.68), 21.00	18.49 (5.44), 19.00	<.001

PSS = Perceived Stress Scale

With respect to the third objective, all regression models were significant. Attachment anxiety predicted perceived stress (β = .504, *t* = 8.188, *p* < .001) and so did attachment avoidance (β = .237, *t* = 3.935, *p* < .001). Both dimensions of insecure attachment explained 36.8% of the variance (adjusted R^2^ = .368).

**Figure 1:**
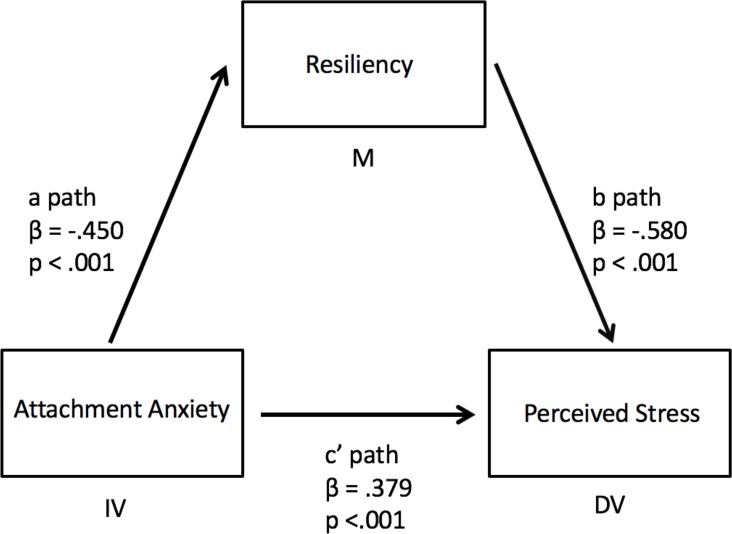
Partial mediation of the relationship between attachment anxiety and perceived stress by resiliency

**Figure 2:**
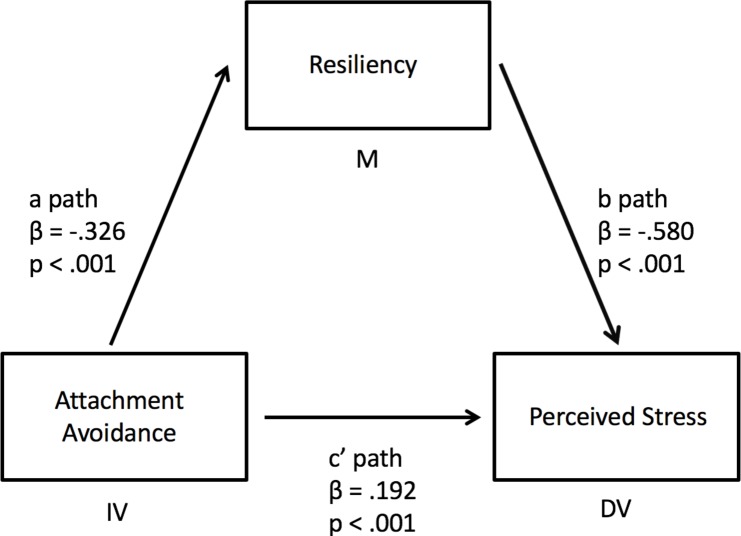
Partial mediation of the relationship between attachment avoidance and perceived stress by resiliency

To address objective four, we conducted univariate analyses between all variables. As all univariate analyses were significant (all *p* < .001), we then conducted the mediation analyses. Following the addition of resilience as a mediator between attachment anxiety and perceived stress, that relationship remained significant, although less so, with an associated change in the Beta value (*p* < .001, △β = -.184; Figure 1). This indicated that resilience is a partial mediator of the relationship between attachment anxiety and perceived stress. Similar results were found when attachment avoidance was used as the independent variable (△β = -.169, Figure 2).

## Discussion

The overall purpose of this project was to increase our understanding of psychological vulnerabilities (i.e., individual attachment) as well as possible protective factors (i.e., resilience) present in medical students, and their role in the level of perceived stress. Our results suggest the medical students have higher prevalence of insecure attachment styles (50.6%) and lower rates of secure attachment styles (49.4%) when compared to previously published norms from the general population (41% vs 59% respectively).^31^ Moreover, the rate of fearful attachment (13.6%) appeared higher in our sample of medical students compared to the general population (4.5%).^31^ Our results are in keeping with a recent meta-analysis of data from US college students, describing a trend towards a greater prevalence of insecure attachment in undergraduate students, with a mean of 58.3% insecure attachment and 41.6% secure attachment.^20^ However, two studies of US medical students suggest lower rates of insecurity and higher rates of secure attachment than found in our study. For instance, when exploring the relationship between attachment and specialty choice in US medical students, Ciechanowski et al. found that almost 60% of medical students (56% and 59%) reported secure attachment in two studies.^32,33^ Even though both studies found the same level of attachment security, the attachment distribution could not be explained by the variation in gender distribution (one study had significantly more male subjects while the other had significantly more females). Similar to our study, Ciechanowski et al., used the same categorical measure for attachment (RQ), allowing for easier comparison. Interestingly, both of these studies identified significantly higher percentages of married or living as married students (57% and 31%) compared to our sample (11%). This might suggest that medical students in committed relationships might benefit from protective support and intimacy, which are likely to alleviate insecure attachment These findings are consistent with recent research showing the healing role played by positive, intimacy-promoting relationships on attachment insecurity.^34^

With respect to our second objective, we found that female students reported significantly higher attachment anxiety than males. This is the first study to demonstrate a gender difference in the percentage of insecurely attached medical students. Similar to our findings, an international meta-analysis showed that females generally exceed males in attachment anxiety though this gender difference was heterogeneous and typically small in college student samples.^19^ In addition, our results showed that females were also more likely to report higher levels of perceived stress and lower resilience. Similar findings were reported by Rahimi et al., who also showed higher levels of perceived stress and lower resilience in female medical students.^2^

The examination of our third objective revealed that attachment insecurity was strongly associated with the level of perceived stress. The importance of this finding can be understood in the context of previous literature which has shown that the level of perceived stress predicts the development of depressive and anxiety symptoms, therefore suggesting a cycle of vulnerability.^35^ When examining the relationship between attachment and the perceived level of stress, several interpretations seem plausible. Pre-existing insecure attachment might impact the ability to cope and maintain a balanced life when faced with school pressures, which in turn can impact the perception of the level of stress. This is also in keeping with previous literature suggesting higher level of stress reported by individuals with preoccupied and fearful attachment.^36^ Conversely, the high paced medical training could impact the perception and expression of attachment security in close relationships, given the demands of energy and time commitment, likely to impact personal life and therefore close relationships. These reciprocal influences fit with previous understanding of attachment as having both trait and state components. For instance, while attachment has been overall regarded as moderately stable through life (the trait component), the impact of various life situations on attachment has also been acknowledged (the state component).^37,38^ Moreover, our data collection was timed at the beginning of the academic year (August-September) which might also fail in capturing the full level of stress associated with prolonged exposure to school stressors and exam periods.

As hypothesized in our final objective, resilience was a partial mediator of the relationship between attachment and the level of perceived stress. This is somewhat intuitive, as resilience refers to the ability to thrive in the face of stress or adverse events. This also suggests a possible avenue for preventive interventions, since resilience has been shown to be amenable to training.^39,40^ Still, while promising, current literature looking at resilience training in medical professions lacks rigour and shows inconsistent results.^41^ For instance, in medical students, a cross-sectional study found that self-compassion and mindfulness training were related to higher resilience and less burn-out symptomatology in medical students.^42^ Still, other studies have found that the programs meant to increase resilience in medical residents were generally feasible and favourably received, but did not lead to statistically significant improvement.^43,44^ Similarly, secure-attachment priming can improve measures of mental health.^10^ Overall, these findings suggest that resilience as well as attachment might be amenable to interventions. However, the refinement of these interventions and the evidence for their efficacy in medical training still require attention. While attachment and resilience describe psychological characteristics of the individual, implicated in cultural adaptation, the reverse influence that culture has on the individual cannot be ignored. In fact, recent scientific debates are considering a contextual understanding of attachment, by moving away from interpreting the research findings as universally applicable across cultures, to an understanding that sees the influence of culture on family values, including parenting, education, and standards of health.^45^ It is, therefore, possible that environments focused on achievement, individuality and competitiveness, might be more prone to aggravating attachment insecurity, inducing more work-related burnout and less relational health. Of note a recent review offers several considerations for improving medical student well-being by modifying curricula or providing skills training, while also noting the need for more research in this area.^46^

### Limitations

We acknowledged several limitations of this study. The cross-sectional design of this study precludes interpretations regarding causality. Though participant response rate was adequate, we surveyed only one medical school, so our results may not be generalizable to other programs in Canada or the US.

### Strengths

The study benefited from a good sample size and measures with acceptable reliability. The study targets a very important area pertaining to the health and psychological vulnerabilities as well as psychological strengths of physicians-in-training. This study is a practical response to the call to improve wellbeing in medical students and to consider attachment and resilience as useful paradigms when designing future supportive interventions.^6,46,47^

### Conclusions

Medical students face unique training and psychological challenges, requiring better understanding and support. Attachment insecurity and resilience appear to play important roles in the level of perceived stress, particularly in females. Despite some of the inherent stressors of training in medical schools, understanding these individual vulnerabilities and protective factors might aid in designing interventions that enhance the student’s resilience, alleviate attachment insecurity and promote wellbeing. As medical schools continue to implement supports and programming empiric validation is necessary, where attachment style and resilience may prove valuable subjects for further study.
